# Quality Assurance of Total Carotenoids and Quercetin in Marigold Flowers (*Tagetes erecta* L.) as Edible Flowers

**DOI:** 10.1155/ijfo/3277288

**Published:** 2025-01-15

**Authors:** Kusumiyati Kusumiyati, Ine Elisa Putri, Yuda Hadiwijaya, Agitha Kartika, Yusuf Eka Maulana, Wawan Sutari

**Affiliations:** ^1^Master Program of Agronomy, Faculty of Agriculture, Universitas Padjadjaran, Sumedang, Indonesia; ^2^Laboratory of Horticulture, Faculty of Agriculture, Universitas Padjadjaran, Sumedang, Indonesia; ^3^Department of Agronomy, Faculty of Agriculture, Universitas Padjadjaran, Sumedang, Indonesia

## Abstract

Marigold flowers, which are also known as *Tagetes erecta* L., are widely recognized for their bright colors and health benefits. Therefore, the purpose of this research was to investigate the quality of total carotenoid content (TCC) and quercetin in marigold flowers, specifically the edible ones, using visible near-infrared spectroscopy (Vis-NIRS) technology. Both bioactive compounds in these flowers were assessed using Vis-NIRS. The technology is considered to be a rapid and non-destructive method for evaluating the quality of agricultural products. To ensure accuracy in the evaluation, Vis-NIRS was validated against conventional methods such as spectrophotometry and chromatography. The results showed that the use of Vis-NIRS helped to accurately determine the levels of TCC and quercetin in marigold flowers. Based on the analysis results, the best accuracy values for TCC were as follows: the correlation coefficient of the calibration set (*R*_cal_) was 0.94, the root mean square error of the calibration set (RMSEC) was 125.57, the correlation coefficient of the prediction set (*R*_pred_) was 0.87, the root mean square error of the prediction set (RMSEP) was 129.35, and the ratio of prediction to deviation (RPD) was 2.77. For quercetin, the best accuracy values were: *R*_cal_ was 0.96, RMSEC was 1.63, *R*_pred_ was 0.88, RMSEP was 2.37, and RPD was 2.09. These results demonstrate that the method provides a reliable alternative to destructive method. The research showed that Vis-NIRS served as a reliable quality assurance tool for assessing bioactive compounds in marigold flowers.

## 1. Introduction

Marigold flowers are well-known ornamental plants that attract attention from many people due to their vivid colors and health benefits. These flowers are scientifically called *Tagetes erecta* L., belonging to the Asteraceae family, and these flowers come in several beautiful shades of colors, including orange, yellow, red, and brown, to mention but a few across the varieties. Due to the different colors, marigold flowers make it easier to use in gardens, landscapes, and decorated pots; hence, it can be concluded that it has a big role in enhancing the beauty of the surrounding environment. Besides being attractive and beautiful, marigold is also an edible flower, used especially in culinary. Besides culinary uses, marigold flowers are utilized for the extraction of lutein and zeaxanthin for lutein supplements. These nutrients are generally consumed to prevent age-related macular degeneration.

One of the flowers that appears to possess a rich taste, marigold flowers are classified as ornamental plants as well as delightful garnishes on the culinary map. Edible flowers such as marigolds are used in preparing salads, desserts, and drinks to improve their appearance and taste. Besides, these flowers possess quality components, especially secondary metabolites that have health implications for humans. Phytochemicals in marigold flowers, which are related to the secondary metabolites of the plant, may also be involved, depending on the geographic origin of the flower and growing conditions such as the weather, light, temperature, soil, and fertilizers [1].

The secondary metabolites present in the marigold flowers, the group of total carotenoid content (TCC), including lutein and zeaxanthin, have outstanding importance [2]. It is important to note that these compounds are critical in the health of the eyes, and therefore, the use of marigolds offers a measure towards control of age-related eye diseases [3]. In addition, marigolds contain flavonoids, a class of antioxidants that are crucial in combating radicals from inside the human body. Flavonoids like quercetin have been shown to have high antioxidant capacity, which can be beneficial to humans [4]. Determining the quality of marigold flowers through laboratory analysis is crucial to identifying and evaluating their components. One widely used method in the analysis involves the extraction of active compounds [5], which provides comprehensive information on flower quality. Through sample extraction, laboratory analysis can identify and quantify secondary metabolites, such as flavonoids and TCC. This evaluation provides an in-depth understanding of the antioxidant and anti-inflammatory properties inherent to marigold flowers, with potential health benefits for humans.

Quality analysis is essential for ensuring the reliability of products intended for market use. In this case, destructive method is usually performed to determine the quality of marigold flowers by extracting specific constituents. In general, destructive analysis has some limitations. Firstly, it causes damage to the analyzed marigold flower samples, which may distort the cell structure. Additionally, the flowers may lose their volatile compounds, which contribute to their distinctive aroma. Secondly, there is a genuine possibility that the chemical composition may be altered during the analysis if the active compounds degrade or oxidize. Thirdly, laboratory analysis is both a lengthy and costly process that requires complex equipment and skilled personnel, making routine or large-scale testing challenging [6, 7]. In order to overcome these obstacles, a non-destructive method known as visible near-infrared spectroscopy (Vis-NIRS) technology may be applied in order to effectively determine the quality of marigold flowers. Besides the rapidity and simplicity of the proposed method relative to conventional extraction, spectrophotometry, and chromatography techniques, it is crucial to highlight that Vis-NIRS is also more environmentally sustainable, as it obviates the necessity for dangerous chemicals. This technology provides substantial benefits, including decreased analysis duration, reduced costs, and enhanced accessibility, making it an appropriate choice for routine analysis. Moreover, although the method is economical for regular analysis, the initial cost of equipment and calibration development can be substantial, presenting a further limitation to its wider accessibility.

Vis-NIRS provides an innovative solution for assessing the quality of marigold flowers, specifically edible flowers. This technology enables non-destructive sample analysis [8–12], thereby presenting considerable benefits. In the context of marigold flowers, Vis-NIRS can measure the quality efficiently by offering real-time insights into secondary metabolites, such as flavonoids and TCC [13, 14]. In contrast to conventional laboratory methods, Vis-NIRS is fast and more cost-effective, empowering producers or analysts to monitor flower quality in real time. This facilitates faster decision-making within the supply chain or research context.

The capability of Vis-NIRS to simultaneously measure quality parameters is an added advantage. Portable versions of the technology further enhance its utility by allowing on-site assessment of marigold flower quality directly at the production site, while fourier transform infrared spectroscopy has been used for spectral characterization in *Allamanda neriifolia* Hook flowers, and visible near-infrared (Vis-NIR) hyperspectral imaging has been adopted for classifying the floral origin of honey, the use of Vis-NIRS for predicting the quality content of flowers, particularly edible ones, remains unexplored [15, 16]. Previous investigations have successfully used spectroscopy to predict TCC and quercetin content [17–21]. Therefore, this research was aimed at assessing Vis-NIRS as a quality assurance tool for TCC and quercetin in marigold flowers. Using technology not only presents a practical solution for non-destructive quality testing but also paves the way for further investigation and innovation in optimizing the production and utilization of the flowers.

## 2. Materials and Methods

### 2.1. Sample Preparation and Spectra Acquisition

Marigold flowers were harvested at full bloom, sorted, and placed in trays, each assigned a number for easy identification during testing. A total of 40 marigold flower samples were used, comprising 30 samples in the calibration set and 10 samples in the prediction set. Spectral data acquisition was performed using a Vis-NIR spectrometer with a wavelength range of 381–1065 nm at 3 nm intervals (NirVana AG410, Integrated Spectronics Pty Ltd., North Ryde, Australia). Each flower sample was irradiated at three different points (top, middle, and bottom), and their spectral data were then averaged.

### 2.2. Processing of Dried Samples

The fresh marigold flowers were cut into pieces and arranged on a tray. The samples were then dried in an oven at 50°C. After drying, the samples were ground into a powder, placed in ziplock bags, and labeled accordingly with identification numbers.

### 2.3. TCC and Quercetin Analysis

The determination of TCC was conducted using the spectrophotometry method [22, 23]. The powdered samples were weighed at 0.01 g and placed into volumetric flasks. Subsequently, 8 mL of acetone was added, and the mixture was sonicated (Baku BK-2000, Guangzhou, China) for 30 min at room temperature. Acetone was also added to the mark, and the solution was transferred to a centrifuge tube for 10 min at a speed of 4000 rpm. The resulting filtrate was diluted tenfold, and TCC was analyzed using UV-Vis spectrophotometer (Shimadzu, UV-1601, Japan), at a wavelength of 450 nm. *β*-Carotene was used as the standard, and TCC results were expressed in milligrams per 100 grams (mg/100g). Meanwhile, quercetin testing was conducted through the chromatography method, expressed in mg/100g.

The filtrate was placed in a fume hood, and 5 mL of sulfuric acid was added. It was then sonicated at 80°C for 45 min, and ethanol was added. Furthermore, 1 mL of the filtrate was injected into high-performance liquid chromatography (HPLC) (Shimadzu, LC 20AT Prominence, Tokyo, Japan) after roughly 10 mL of the filtrate was centrifuged for 10 min. The following nutrients were determined in each of the extracts, and the result was expressed in mg/100g using the quercetin standard curve. Subsequently, another previously described method, known as the spectrophotometric method, was used to determine the TCC of marigolds [24]. Firstly, 0.5 mL of the sample was pipetted into a 3 mL vial and then mixed with 0.25 mL of 7% H_2_SO_4_. The mixture was then heated at 80°C and incubated for 30 min; thereafter, the volume of the mixture was made up to 1 mL. Lastly, the volume of the solution inside the vial was measured using HPLC. The measurement was done using a mobile phase gradient of 1% acetic acid and acetonitrile (40:60) at a flow rate of 1 mL/min, with a temperature of 28°C, a C18 column (150 × 4.6 mm, 5 *μ*M), a detection wavelength of 272 nm, an injection volume of 20 *μ*L, and an analysis time of 40 min.

### 2.4. Data Analysis

**Figure 1:**
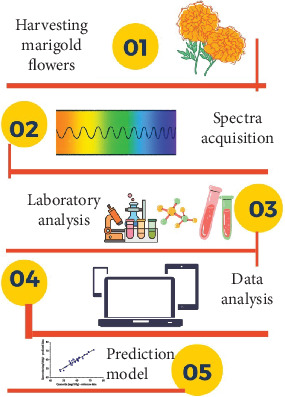
Illustration of Vis-NIRS acquisition of marigold flowers.

All the collected data were analyzed using the Unscrambler X 10.4 software from Camo AS, Oslo, Norway [25]. This work was only related to absorbance data and method development carried out using partial least squares regression (PLSR). In the first instance, the pretreatment used for the removal of spectral shifts comprised standard normal variate (SNV), multiplicative scattering correction (MSC), first derivative of Savitzky–Golay (dg1), second derivative of Savitzky–Golay (dg2), and de-trending. The obtained statistical parameters included the correlation coefficient on the calibration set (*R*_cal_), the root mean square error of the calibration set (RMSEC), the correlation coefficient on the prediction set (*R*_pred_), the root mean square error of the prediction set (RMSEP), as well as the ratio of prediction to deviation (RPD). An illustration of the research process is presented in Figure 1.

## 3. Results and Discussion

### 3.1. Reference Data Obtained by Laboratory Measurements

Reference data for quercetin and TCC measurements in marigold flowers were obtained through laboratory analysis (Table 1). These measurements were crucial as they indicated variations in the data across the samples, assisting the analysts in understanding the data variability. In Table 1, TCC in marigold flowers ranged from 2843.82 to 3998.19 mg/100 g, with an average of 3362.58 mg/100 g and a standard deviation (SD) of 352.48 mg/100 g. The TCC results exceeded those reported by Akshaya et al. [26, 27] and Lohar et al. [28], where they ranged from 19.61 to 2765.76 mg/100 g. As for quercetin, the range was from 47.16 to 72.77 mg/100 g, with an average of 59.98 mg/100 g and an SD value of 6.13 mg/100 g. However, the quercetin content appeared lower compared to previous research by Ingkasupart et al. [29], which reported the content ranging from 615 to 1261 mg/100 g.

### 3.2. Analysis of Diverse Types of Spectra

**Figure 2:**
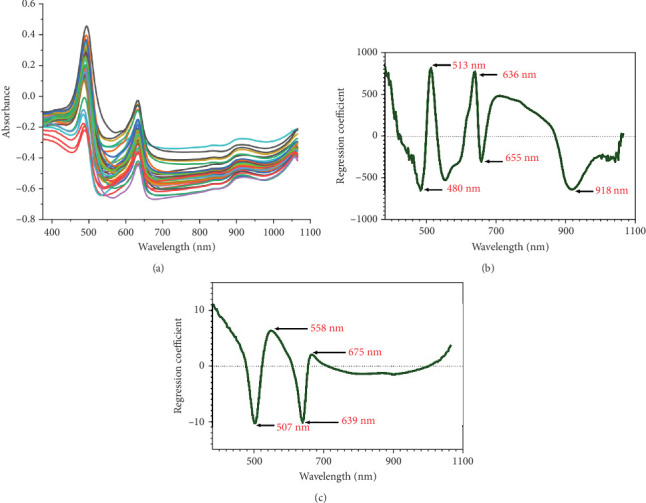
Spectral characteristics of marigold flower: (a) full spectra, (b) regression coefficient from TCC, and (c) regression coefficient from quercetin.

Figure 2 presents the peaks and valleys crucial for evaluating the observed parameter values.  Figure 2(a) visualizes the full spectra during data acquisition using a Vis-NIRS spectrometer, where the *x*-axis signified wavelengths and the *y*-axis showed the absorbance of samples. Peaks and valleys on the spectra describe the sample's response to the light intensity, with peaks representing the highest points on the graph and indicating the presence of specific substances or compounds [24]. When a sample absorbed light at specific wavelengths, peaks appeared in the spectra and it corresponds to the specific substance. Therefore, the full spectra image with peaks and valleys provided a clear visual indication of wavelengths at which a particular substance interacted with light.

Observing peaks and valleys in the full spectra image could provide insights into electronic energy transitions in molecules. Analyzing these features offered detailed information about the composition and characteristics of the sample [30]. Therefore, the full spectra image with peak and valley analysis enhanced the understanding of the chemical nature and molecular structure of the tested sample. A comprehensive examination of the spectra indicates that the observed peaks and valleys indicate the presence of substances and offer insight into the sample's molecular interactions with light. The specific wavelengths relevant for identifying the TCC and quercetin are identified in Figures 2(b) and 2(c). In Figure 2(b), the regression coefficient of TCC showed peaks and valleys at wavelengths including 480, 513, 636, 655, and 918 nm, highly correlated with color pigments and water content [31, 32].  Figure 2(c) presents the regression coefficient wavelengths for quercetin, comprising 507, 558, 639, and 675 nm.

### 3.3. Development of a Calibration Model for Marigold Flower Among Spectra Pretreatment Method

The development of a calibration model for marigold flowers comprised exploring various spectral correction methods to enhance accuracy (Table 2). Each spectra pretreatment method could influence the model differently, indicating its strengths and limitations in improving accuracy. This research found that the original spectra produced higher accuracy values when compared to other methods. For TCC, the accuracy values from the original spectra included *R*_cal_ of 0.94, RMSEC of 125.57, *R*_pred_ of 0.87, RMSEP of 129.35, and RPD of 2.77. The results were comparable to those obtained by Toledo-Martín et al. [33], who achieved similar accuracy values for TCC parameters in blackberry fruit. The highest calibration model for quercetin was obtained from the original spectra, with *R*_cal_ of 0.96, RMSEC of 1.63, *R*_pred_ of 0.88, RMSEP of 2.37, and RPD of 2.09. The results for both TCC and quercetin parameters were categorized as good predictions, characterized by low RMSEC and RMSEP, high *R*_cal_/*R*_pred_ values, and minimal discrepancy between RMSEC and RMSEP [34–36].

**Figure 3:**
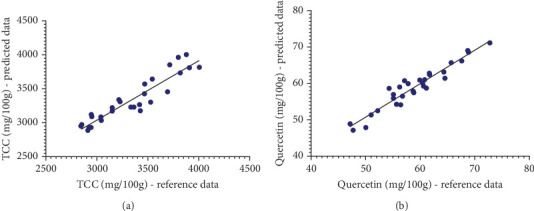
Scatter plot of calibration model on marigold flowers: (a) TCC and (b) quercetin.

A scatter plot visually represented the relationship between reference data and predicted data from the analysis of TCC and quercetin levels in marigold flowers, aiding analysis in understanding distribution, trends, and correlations. Each point on the graph corresponded to one sample, with the *x*-axis typically showing reference data for the observed parameter and the *y*-axis indicating the predicted results by Vis-NIRS. This allowed the scatter plot to clearly indicate the relationship between the two variables. As shown in Figure 3, the resulting scatter plot displays the distribution of TCC and quercetin concentration data for each sample, revealing any patterns or variations within the dataset. Points that fall close to the regression line on the scatter plot indicate a strong relationship between the two observed variables [37, 38].

The regression line shows the statistical relationship between variables, where points clustered closely together signify that the model effectively captures the pattern or trend in the data. The proximity of the points to the regression line demonstrates how the reference value and the forecasted values are related. The points are closely distributed around the regression line, and the relationship between the variables becomes stronger, indicating a more accurate regression model. Analyzing points that are closely correlated with the regression line provides further confidence in the validity of the model. By examining how closely these points follow the trend of the regression line, the analyst gains confidence in determining the reliability of the model in predicting the observed parameters. Therefore, the closer the points are to the regression line, the higher the accuracy of the regression model.

## 4. Conclusions

This research showed the potential of Vis-NIRS as an alternative technology for predicting TCC and quercetin in marigold flowers. Several spectra pretreatment methods, including MSC, SNV, dg1, dg2, and de-trending, were incorporated into the calibration model. However, the analysis found that the most precise predictions for TCC and quercetin parameters were obtained using the original data. The best predictions for TCC and quercetin resulted in *R*_cal_ ≥ 0.94, *R*_pred_ ≥ 0.87, and RPD ≥ 2.09. This indicated the effectiveness of Vis-NIRS in predicting the content of TCC and quercetin in marigold flowers. The technology offered significant benefits, particularly for analysts or industry professionals, enabling them to assess marigold flower content without resorting to destructive methods. Marigold flowers with acquired quality data could still be marketed, providing consumers with product assurance while producers avoided losses resulting from destruction. Therefore, this technology was expected to significantly contribute to non-destructive method, particularly in agricultural field.

## Figures and Tables

**Table 1:** Reference data for TCC and quercetin in calibration and prediction set.

**Quality attributes**	**Minimum**	**Maximum**	**Mean**	**SD**
TCC (mg/100 g)	2843.82	3998.19	3362.58	352.48
Quercetin (mg/100 g)	47.16	72.77	59.98	6.13

Abbreviations: SD: standard deviation, TCC: total carotenoid content.

**Table 2:** Comparison of accuracy of different spectrum pretreatment methods for TCC and quercetin.

**Quality**	**Spectra**	**R**_**c** **a** **l**_	**RMSEC**	**R**_**p** **r** **e** **d**_	**RMSEP**	**RPD**
TCC	**Original**	**0.94**	**125.57**	**0.87**	**129.35**	**2.77**
	MSC	0.93	128.87	0.77	182.29	1.96
	SNV	0.93	134.43	0.77	182.33	1.96
	dg1	0.94	124.10	0.77	180.53	1.98
	dg2	0.95	111.47	0.55	250.85	1.43
	Detrending	0.95	115.61	0.85	140.58	2.55

Quercetin	**Original**	**0.96**	**1.63**	**0.88**	**2.37**	**2.09**
	MSC	0.91	2.60	0.82	2.95	1.93
	SNV	0.91	2.51	0.85	2.57	1.93
	dg1	0.93	2.28	0.84	3.90	1.27
	dg2	0.90	2.67	0.72	4.58	1.08
	Detrending	0.85	3.21	0.86	3.13	1.59

*Note:* The data presented in bold describe the best model for each quality attribute.

## Data Availability

The data that supports the findings of this study are available from the corresponding author upon reasonable request.
